# Diagnostic Performance of ChatGPT in Corneal Disease Recognition From Slit-Lamp Photographs

**DOI:** 10.7759/cureus.96161

**Published:** 2025-11-05

**Authors:** Muneeb A Khan, Diya Baker

**Affiliations:** 1 General Practice, Royal Stoke University Hospital, Stoke on Trent, GBR; 2 Ophthalmology, Royal Wolverhampton NHS Trust, Wolverhampton, GBR

**Keywords:** artificial intelligence, chatgpt, cornea, large language model, ophthalmology, slit-lamp photography

## Abstract

Aim: To evaluate the diagnostic accuracy of ChatGPT-4o (OpenAI San Francisco, CA, USA), a large language model (LLM), in identifying corneal pathology solely from slit-lamp photographs, without any additional clinical context, and compare accuracy to consultant ophthalmologists.

Methods: This was a prospective diagnostic accuracy study. A total of 22 images were selected from the Atlas at EyeRounds.org (The University of Iowa). Diagnostic accuracy, defined as the proportion of correctly identified cases, was calculated for ChatGPT-4o and two consultant ophthalmologists, against the reference standard. Pairwise comparisons between ChatGPT-4o and each consultant ophthalmologist were performed using McNemar’s test to evaluate differences between the AI model and each consultant ophthalmologist.

Results: The accuracy of identifying a characteristic sign or diagnosis for ChatGPT was 0.50 (95% CI: 0.28 - 0.72, p-value 1.00), compared to 0.64 (95% CI: 0.41 - 0.83, p-value 0.29) for Ophthalmologist A and 0.55 (95% CI: 0.32 - 0.76, p-value 0.83) for Ophthalmologist B. McNemar’s test demonstrated a statistically significant difference between ChatGPT and Ophthalmologist A (p = 0.01), whereas no statistical significance was observed between ChatGPT and Ophthalmologist B (p = 0.37).

Conclusions: In this study, ChatGPT-4o demonstrated moderate diagnostic accuracy in identifying corneal pathology from slit-lamp photographs, with performance comparable to that of consultant ophthalmologists. These findings highlight the potential feasibility of using LLMs as adjunctive tools in ophthalmic image interpretation. Limitations include the AI model’s tendency to produce confident yet occasionally inaccurate responses. While not yet suitable for autonomous diagnostic use, ChatGPT-4o shows promise as a supportive aid in clinical decision-making when used under appropriate expert supervision.

## Introduction

Artificial intelligence (AI) has shown strong potential in ophthalmology, with deep learning models achieving over 85% sensitivity and specificity in diabetic retinopathy detection, leading to FDA-approved autonomous screening systems [1].

Traditional deep learning models in ophthalmology are task-specific and limited to single-modality inputs, typically images. Recent advances in large language models (LLMs) with multimodal capabilities, such as GPT-4o (OpenAI San Francisco, CA, USA), introduce a framework for simultaneous processing of visual and textual information, enabling preliminary applications in clinical imaging, including ophthalmic photographs, although diagnostic performance remains variable and limited when compared with human experts [2,3].

This study presents an evaluation of ChatGPT-4o’s ability to correctly diagnose or identify a key finding of corneal pathology against the reference standard of the image bank, solely from slit-lamp photographs, without any additional clinical context. The aim is assessment of diagnostic accuracy, and comparison of results to consultant ophthalmologists, based on standardised prompts and isolated image input.

## Materials and methods

This was a prospective diagnostic accuracy study evaluating the performance of a large language model (ChatGPT-4o) in identifying corneal pathology from slit-lamp photographs. A total of 22 anonymised images were selected from the publicly available Ophthalmic Atlas at EyeRounds.org (The University of Iowa), used exclusively for the purpose of this research. Inclusion criteria required that each image display a characteristic clinical sign or feature of a single, identifiable corneal pathology. Images were excluded if they involved non-corneal pathology, multiple diagnoses, foreign bodies, or evidence of surgical intervention (e.g., implants or sutures). Case notes associated with each image were reviewed to ensure appropriate selection and diagnostic clarity.

Each image was renamed using a numerical identifier from 1 to 22 and uploaded individually into the ChatGPT interface (ChatGPT Plus subscription, GPT-4o model). A standardised prompt was used for each image, requesting identification of the most characteristic sign and/or likely corneal diagnosis. The model was instructed not to use external data sources such as an internet search or utilising other databases, to restrict interpretation to corneal pathology only from the image and associated prompt. The model was also tasked to comment on any image artefacts or resolution limitations that might impair analysis.

For reference standard comparison, the documented diagnosis or characteristic sign provided by EyeRounds.org for each image was used. ChatGPT responses were independently assessed and scored as correct or incorrect based on concordance with these reference diagnoses. The same anonymised image set was also presented to two consultant ophthalmologists, who were similarly asked to provide the characteristic sign and/or most likely diagnosis for each image without access to additional clinical information. Their responses were scored using the same criteria.

Diagnostic accuracy, defined as the proportion of correctly identified cases, was calculated for ChatGPT and each of the two consultant ophthalmologists. Pairwise comparisons between ChatGPT and each Consultant Ophthalmologist were performed using McNemar’s test to evaluate differences in classification accuracy. Statistical significance was defined as p < 0.05.

## Results

Diagnostic performance was evaluated across 22 clinical cases, comparing ChatGPT to two ophthalmologists (A and B). The accuracy of identifying a characteristic sign or diagnosis for ChatGPT and each consultant ophthalmologist is given in Table 1. McNemar’s test was performed to assess pairwise differences in diagnostic accuracy. The results of this are given in Table 2. ChatGPT provided a unique correct diagnosis in one case (Pseudomonas keratitis) that neither ophthalmologist identified. Conversely, there were five cases where both ophthalmologists correctly identified the diagnosis while ChatGPT did not.

**Table 1:** Diagnostic accuracy and 95% confidence intervals for ChatGPT and two ophthalmologists in identifying the presence of a characteristic sign or diagnosis using 22 cases

Assessor	Correct	Total	Accuracy	p-value	95% CI (higher)
ChatGPT	11	22	0.50	1.00	0.28 - 0.72
Ophthalmologist A	14	22	0.64	0.29	0.41 - 0.83
Ophthalmologist B	12	22	0.55	0.83	0.32 - 0.76

**Table 2:** McNemar’s test results comparing ChatGPT to two ophthalmologists

Comparison	Both Correct	Both Incorrect	Ophthalmologist only Correct	ChatGPT only Correct	McNemar’s Test Statistic	p-value
ChatGPT vs Ophthalmologist A	7	5	9	1	6.40	0.01
ChatGPT vs Ophthalmologist B	6	5	7	4	0.82	0.37

## Discussion

The results indicate that ChatGPT-4o’s diagnostic performance was modest but within the range of human experts. McNemar’s test showed statistically significant differences between the LLM and one out of two clinicians. These findings suggest that ChatGPT’s image-based diagnostic ability is broadly comparable to that of practicing ophthalmologists under the specific conditions tested. This aligns with prior studies indicating that GPT-based models can approach clinician-level performance on complex diagnostic tasks [2,4]. For example, Sorin et al. reported that multimodal ChatGPT-4 (GPT-4V) correctly diagnosed 47.5% of cases using ocular images alone, improving to 67.5% when provided with clinical context [2]. This image-only accuracy is similar to the 50% rate achieved by ChatGPT-4o in our study, despite differences in types of cases and model versions. Likewise, another study utilising GPT-4V found correct identification on slit-lamp photographs to be 42%, higher than the results obtained for optical coherence tomography, fundus fluorescein angiography and ocular ultrasound images [3].

Dedicated deep learning algorithms trained on large slit-lamp datasets achieve significantly higher diagnostic accuracy in corneal disease than current LLMs [5,6]. These task-specific convolutional neural networks (CNNs) benefit from training on large, labeled image sets and consistently outperform general-purpose models in image-only classification tasks. However, these CNNs are typically optimised to deliver discrete outputs, class labels or probability scores and do not inherently generate explanatory text. Any narrative reasoning, differential generation, or clinical interpretation must be added separately by human experts or through additional software. This limitation constrains their use in clinical decision-making beyond classification. Additionally, the implementation of such domain-specific models requires substantial computational infrastructure and training datasets, conditions that are largely concentrated in high-resource academic or commercial centres. As a result, deployment of CNN-based systems is not yet uniformly accessible across regions. However, domain-trained AI is likely to remain more accurate for pure image classification.

By contrast, ChatGPT, a generative LLM, is broadly accessible, user-friendly, and rapidly evolving. It can be used “off the shelf” without any prior model training, to generate free-text outputs directly from a prompt. When provided with a single image or a combination of image and clinical context, ChatGPT can produce structured, human-like reasoning and incorporate differential diagnosis and rationale, mirroring the approach taken by a clinician. This capacity to integrate inputs and generate narrative explanations gives LLMs potential for suitability in augmenting clinical workflows, particularly in settings lacking domain-specific AI systems. For these reasons, ChatGPT-4o was the model evaluated in our study.

Notably, ChatGPT correctly identified one case of Pseudomonas keratitis that both consultants missed. This diagnosis was attained despite no specific prompt given to any assessor to attempt subcategorisation of microbial keratitis. The LLM may have leveraged generalised patterns learned from text, such as an association of a “green infiltrate” with Pseudomonas, that the consultants overlooked. In contrast, the LLM performed poorly across all corneal dystrophy images with the exception of Map-dot-fingerprint dystrophy, highlighting a likely difficulty in delineating certain subtle differences in texture or patterns of arrangements associated with certain pathological findings.

A further concern lies in the presentation of ChatGPT’s output. Like other large language models, it can produce fluent and persuasive but incorrect responses. Prior analyses have shown that this fluency may lead users to overestimate the accuracy of its answers [7]. In a clinical setting, this creates the risk that without expert oversight, incorrect but confidently stated interpretations could go unchallenged. Human clinicians, by contrast, apply critical judgment, weigh differential diagnosis and consider contextual information. These are functions that ChatGPT currently appears to lack.

Given these findings, ChatGPT-4o remains far from a viable autonomous diagnostic tool; for now it is best positioned as a decision-support aid, for example, generating differentials or highlighting salient features, and serving as a ‘second opinion’ in resource-limited settings. Any clinical deployment must involve human supervision, with clinicians verifying outputs against their own interpretation and patient data. Human expertise remains indispensable for integrating visual, historical, and contextual information to guide accurate clinical decision-making.

Despite these constraints, recent developments in model training and prompt engineering may enhance ChatGPT’s image interpretation capabilities over time. Further validation studies could test whether disease-specific, texture-oriented prompts improve detection. Future work could enhance performance by combining ChatGPT with specialised image-analysis models. For instance, CNNs could first segment or classify key features, then feed textual outputs into ChatGPT for broader interpretation. As models evolve, their capacity to correctly recognise the salient features of medical images should grow. However, domain-trained AI is likely to remain more accurate for pure image classification.

Several limitations must be considered when interpreting the results. The AI model we tested primarily processes textual information and has limited specialised visual processing capabilities. By using 22 images from one image atlas, we have not utilised the full spectrum of a pathological presentation. Comparisons in our study only involved two ophthalmologists, limiting generalisability. Additionally, we utilised a relatively small sample size, which limits statistical power.

## Conclusions

In this study, ChatGPT-4o demonstrated moderate diagnostic accuracy in identifying corneal pathology from slit-lamp photographs, with performance comparable to that of consultant ophthalmologists under image-only conditions. These findings highlight the potential feasibility of using LLMs as adjunctive tools in ophthalmic image interpretation. However, limitations remain, particularly the AI model’s tendency to produce confident yet occasionally inaccurate responses. While not yet suitable for autonomous diagnostic use, ChatGPT-4o shows promise as a supportive aid in clinical decision-making when used under appropriate expert supervision.
